# Altruism Enhances Frontal Alpha Asymmetry and Reduces Sympathetic Activity: A Multimodal EEG‐ECG Study With Implications for Therapeutic Interventions

**DOI:** 10.1002/brb3.70747

**Published:** 2025-08-21

**Authors:** Junya Orui, Keigo Shiraiwa, Takao Inoue, Masaya Ueda, Keita Ueno, Yasuo Naito, Ryouhei Ishii

**Affiliations:** ^1^ Department of Health Science Osaka Health Science University Osaka Japan; ^2^ Department of Occupational Therapy Osaka Metropolitan University Graduate School of Rehabilitation Science Habikino Japan; ^3^ Department of Rehabilitation Osaka Kawasaki Rehabilitation University Kaizuka Japan; ^4^ Department of Psychiatry Osaka University Graduate School of Medicine Suita Japan

**Keywords:** altruism, autonomic activity, electroencephalography, frontal alpha asymmetry, neurophysiology, social cognition, therapeutic interventions

## Abstract

**Introduction:**

This study aimed to investigate neurophysiological differences between altruistic and selfish behaviors by simultaneously measuring electroencephalography (EEG) and electrocardiography (ECG). Specifically, we hypothesized that altruistic behavior would be associated with distinct patterns of cortical activity and autonomic responses.

**Methods:**

Thirty‐one healthy participants (17 females; mean age: 20.00 ± 1.18 years) completed crafting tasks in a counterbalanced order under altruistic and selfish conditions. We measured and analyzed frontal alpha asymmetry (FAA) scores, cardiac sympathetic index (CSI), and cardiac vagal index (CVI). Additionally, we used eLORETA (exact‐low resolution electromagnetic tomography) to examine current source density and functional connectivity patterns across brain regions.

**Results:**

The altruistic condition exhibited significantly higher FAA scores (*p* = 0.031, *r* = 0.45) and lower CSI (*p* = 0.048, Cohen's *d* = 0.37) compared to the selfish condition. Notably, novel correlations were observed between neurophysiological measures and specific brain regions. Specifically, FAA scores were associated with gamma activity in the anterior cingulate cortex during the altruistic condition (*p* = 0.071) and with precuneus activity during selfish behavior (*p* = 0.029). Additionally, distinct functional connectivity patterns were associated with autonomic activity in the altruistic condition. Parasympathetic activity negatively correlated with temporal‐gamma connectivity (*p* = 0.002), and heart rate change negatively correlated with temporal‐prefrontal theta connectivity (*p* = 0.048).

**Conclusions:**

Our findings reveal the intricate relationship between cortical activity, functional connectivity, and autonomic responses during altruistic versus selfish behaviors for the first time. This integrative approach sheds new light on the neural mechanisms underlying social cognition. This approach also has the potential to enhance our understanding of and ability to encourage prosocial behavior in various clinical and therapeutic settings.

## Graphical abstract

The study reveals distinct neurophysiological patterns associated with altruistic and selfish behaviors, including differences in frontal alpha asymmetry, cardiac indices, and functional connectivity. These findings provide novel insights into the neural mechanisms underlying social cognition and prosocial behavior.

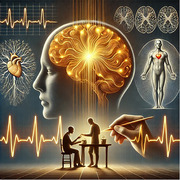

## 1 Introduction

Altruism is a fundamental aspect of human society, crucial for social cohesion and individual well‐being. Altruism is defined as an apparently unselfish behavior that provides benefit to others at some cost to the individual (American Psychological Association 2018) and has captivated scholars across diverse disciplines, from evolutionary biology to psychology and sociology. This noble concept contrasts sharply with selfish behavior, which prioritizes one's own interests over those of others. Altruistic acts benefit not only their recipients but also the giver. These benefits include fostering interpersonal bonds, reducing aggressive tendencies, and promoting mental well‐being (Stocks and Lishner 2023). Indeed, altruism emerges as a cornerstone of human existence, essential for our species to flourish within the complex social structures (Rahimyar and Sarvari 2023). The therapeutic potential of altruism has been increasingly recognized, with empirical evidence suggesting its efficacy in promoting psychological welfare (Elsherbiny 2022; L. Li et al. 2023). However, the neurophysiological mechanisms are not fully understood. Advancing our knowledge of social behavior and developing targeted interventions in fields such as rehabilitation and clinical psychology require a better grasp of these mechanisms.

Recent advancements in neuroscience have enabled detailed investigations into the relationship between the brain and behavior. Although functional magnetic resonance imaging (fMRI) research has revealed brain regions associated with altruistic behavior—such as those involved in reward processing and social cognition (Boccadoro et al. 2021; Cutler and Campbell‐Meiklejohn 2019)—the technology's limited temporal resolution prevent us from fully understanding of dynamic neural processes. However, these studies have provided valuable insights into the spatial localization of altruistic processes. They revealed that regions such as the ventral striatum, the anterior cingulate cortex (ACC), and the medial prefrontal cortex (mPFC) are involved in these processes.

In contrast, studies using electroencephalography (EEG) have revealed associations between altruistic behavior and frontal alpha asymmetry (FAA) (Huffmeijer et al. 2012), frequency‐specific brain activity (Rodrigues et al. 2015), and event‐related potentials (Gan et al. 2022; Huang et al. 2021; Luo et al. 2019). The high temporal resolution of EEG allows for the detection of these associations. Thus, EEG is well‐suited for capturing dynamic neural activity associated with altruistic behavior. One key EEG metric is FAA, which reflects the balance of activity between the left and right frontal lobes. Higher FAA scores indicate greater activity in left‐frontal lobe, which has been linked to positive emotions and approach motivation (Harmon‐Jones and Allen 1997; Coan and Allen 2004; Shangguan et al. 2023). Furthermore, EEG functional connectivity (FC) studies have revealed that the altruistic decisions are associated with right‐lateralized empathy networks (Mitiureva et al. 2024).

Heart rate variability (HRV) complements EEG measurements by indicating the activity of autonomic nervous system. HRV reflects the balance between the sympathetic and parasympathetic systems and serves as an indicator of emotional states, such as stress or compassion. Studies have shown that compassion, the basis of altruistic behavior, is linked to parasympathetic dominance and slower heart rate (Eisenberg et al. 1989; Correa et al. 2015; Stellar et al. 2015). However, no study has examined cortical dynamics, FC, and autonomic regulation simultaneously during therapeutic tasks performed with altruistic intent in an ecologically valid setting. This gap is particularly notable given the growing emphasis on neuroscience‐informed interventions in clinical practice. Previous research (Orui et al. 2025) has shown that FAA scores increase during altruistic activities, indicating greater left frontal activity and decreased sympathetic nervous system activity. These findings were based on EEG measurements of the frontal region and autonomic nervous system assessments. Despite these advances, existing research has several limitations. First, most studies have focused on single neurophysiological measures, failing to capture the complex interplay between cortical activity, FC, and autonomic responses. Second, the ecological validity of many experimental paradigms has been questioned because they often involve artificial laboratory tasks that may not accurately reflect real‐world altruistic behaviors. Lastly, limitations in the neuroimaging techniques used in previous studies have obscured the temporal dynamics of altruistic decision‐making processes. To achieve a comprehensive understanding of complex mechanisms of prosocial motivation and to develop novel therapeutic approaches for clinical use, an integrated EEG and autonomic approach is required.

This study aims to address these gaps by investigating the neurophysiological differences that occur when performing tasks with altruistic versus selfish motivation. The study will simultaneously collect EEG and electrocardiogram (ECG) data. Our novel approach integrates multiple neurophysiological measures, including FAA scores, whole‐brain activity patterns across frequency bands, FC between brain regions, and autonomic nervous system indicators such as HRV. This comprehensive methodology will enable us to examine the intricate relationships between cortical activity, neural networks, and autonomic responses during these distinct motivational states for the first time. We hypothesized that:

1. engaging in a task with altruistic motivation is associated with higher FAA scores, which reflect positive emotions and an approach motivation;
2. brain regions involved in social cognition and empathy show increased activity during altruistic condition;
3. autonomic responses are characterized by parasympathetic dominance during the altruistic condition;
4. distinct patterns of correlation between brain activity, FC, and autonomic responses differentiate altruistic conditions from selfish ones. These findings provide a more nuanced understanding of the neural basis of social cognition.

This study takes an integrative approach to elucidate the mechanisms underlying prosocially motivated behavior, offering unparalleled insights into its neurophysiological basis. These insights could inform novel therapeutic approaches to promoting prosocial behavior and mental well‐being across various fields.

## 2 Methods

### 2.1 Participants

We recruited rehabilitation students aged 18–23 years for this study. All participants were given written and verbal explanations of the experimental procedure and purpose and provided written informed consent. Exclusion criteria were (1) physical illnesses interfering with task performance, (2) major stressors as defined by DSM‐5 within the past 6 months, (3) psychiatric consultation history within the past 6 months, and (4) currently taking psychotropic medication. All participants were confirmed to be in good mental and physical health. This study was approved by the Research Ethics Committee of the Graduate School of Rehabilitation Science, Osaka Metropolitan University (approval number 2023–217), and conducted in accordance with the Declaration of Helsinki. Participants were notified of their right to withdraw from the experiment at any point. A power analysis using MATLAB (*α* = 0.05, two‐tailed; power = 0.80; effect size *d* = 0.50) determined a required sample size of 32 based on previous neurophysiological studies of altruistic behavior (Orui et al. 2025). We added a 5% margin to account for potential data loss, setting the target sample size at 34.

### 2.2 Procedure

**FIGURE 1:**
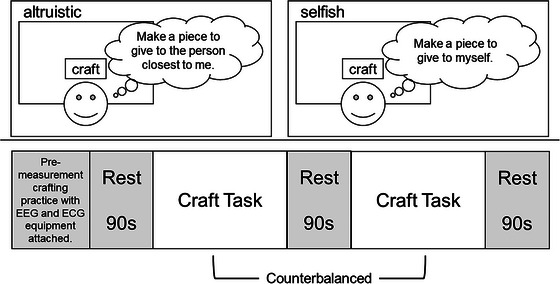
Experimental design and procedure. This schematic illustrates the task paradigm. Participants engaged in a net craft activity under two conditions: altruistic (creating for someone close to them) and selfish (creating for themselves). The order of these conditions was counterbalanced across participants. Each condition was preceded and followed by a 90‐s rest period. Participants completed the task while undergoing simultaneous EEG and ECG recordings.

Participants engaged in a “net craft” task, creating bookmarks by threading a needle through perforated polyethylene canvas, as described in previous research (Orui et al. 2023, 2024). The bookmarks measured 2.4 cm wide by 5.4 cm long, with a canvas containing five horizontal and 12 vertical 3‐mm square holes. The task involved threading through the bottom right hole, moving two places left, and repeating this process 12 times vertically for both left and right columns. Participants performed the task under both altruistic and selfish conditions. In the altruistic condition, they were instructed to create a bookmark for someone close to them, while in the selfish condition, they made one for themselves. To ensure specificity in the altruistic condition, participants were asked to identify their chosen recipient and their relationship to that person prior to the experiment. For ethical reasons, the recipient's gender and age were not controlled. A 90‐s rest period preceded and followed each condition. The order of conditions was randomized to counterbalance potential order effects (Figure 1). Prior to data collection, participants were fitted with EEG and ECG equipment and underwent a practice session for the craft task. This practice continued until they could complete one full bookmark without procedural errors and verbalized confidence in their ability to perform the task. This familiarization phase was designed to minimize potential practice effects during the experimental conditions. Each experimental condition lasted approximately 3–4 min with a 90‐s rest period between conditions. The total experiment time including preparation was approximately 1 h.

### 2.3 Measurement Index

In addition to gender and age, the items measured were as follows.

#### 2.3.1 Task Performance

We measured the time taken to complete the 12‐step, two‐row net craft task to assess potential behavioral effects of motivational differences.

#### 2.3.2 EEG

We recorded EEG activity using a Neurofax EEG‐9100 (Nihon Kohden Corporation) at a 1000 Hz sampling rate. We positioned 19 electrodes according to the international 10–20 system, with electrode resistance maintained below 10 KΩ.

We processed offline EEG data using EEGLAB (v2021.1) on MATLAB, applying bandpass filters (1.5–60 Hz), and utilized Artifact Subspace Reconstruction (ASR) for artifact removal. ASR identified the clean parts of the data, and data exceeding 10 times the standard deviation were rejected using principal component analysis. Independent Component Analysis (ICA) was conducted using the *runica* algorithm, rejecting components identified as electromyography, electrooculography, or electrocardiography with ≥ 90% probability. To minimize movement‐related artifacts, data from the first and last 30 s of each task were excluded from the analysis.

FAA scores were calculated using the EEGLAB *Darbeliai* v2022.12.21.1 plug‐in. We computed power values for F3 and F4 channels (FFT window length: 2 s; spectral step: 10 Hz), calculating the ratio of 8–13 Hz to 1–60 Hz power. FAA scores were derived by subtracting F3 from F4 relative alpha power values, with higher scores indicating greater left frontal activity (Coan and Allen 2004). The use of relative alpha power is supported by previous research demonstrating its higher test‐retest reliability (Fernández et al. 1993; Marshall et al. 2002).

Furthermore, we used exact‐low resolution electromagnetic tomography (eLORETA) (Pascual‐Marqui et al. 2011) to estimate current source density (CSD) and FC of EEG signals. eLORETA can measure neural electrical signals using CSD without requiring a specific number of sources to be active. The resolution is limited to the cortical gray matter and includes 6239 voxels with a 5 mm spatial resolution. After preprocessing to remove artifacts, the EEG data were computed as CSD in five frequency bands: delta (1–3.5 Hz), theta (4–8 Hz), alpha (8.5–13 Hz), beta (13.5–30 Hz), and gamma (30.5–60 Hz). For FC, we used Lagged Phase Synchronization (LPS), a nonlinear FC technique robust to non‐physiological artifacts such as low spatial resolution and volume conduction (Stam et al. 2007). LPS has been effectively applied to filtered EEG data to assess brain FC (Derks et al. 2021). For the FC analysis, we defined 19 anatomical regions of interest (ROIs) based on a well‐established brain network atlas derived from fMRI research (Vincent et al. 2008). It is important to clarify that these 19 ROIs are distinct from the 19 scalp electrodes used for data acquisition; source localization methods (eLORETA) were used to estimate the activity in these underlying cortical regions from the scalp‐level signals. These ROIs include the mPFC, the ACC, and the dorsolateral prefrontal cortex (DLPFC), all of which play an important role in social cognition. While higher‐density EEG systems are preferred for more detailed spatial analyses, 19‐channel EEG with eLORETA has been validated and offers insights in clinical settings. Pascual‐Marqui et al. (2011) showed that eLORETA exhibits zero localization error with fewer electrodes, though spatial resolution is reduced. Recent research has effectively utilized 19‐channel EEG with eLORETA in cognitive and clinical studies (Aoki et al. 2015). Despite spatial resolution limitations, the 19‐channel system provides practical benefits for participant comfort, valuable in clinical applications. We acknowledge the limitations in spatial resolution and have interpreted our results cautiously, using additional EEG metrics and autonomic data to validate our findings.

#### 2.3.3 ECG

We collected ECG data using Polyam (ECG) IIB (Nihon Santeku Co., Ltd.) at a 1000 Hz sampling rate. Electrodes were placed on the upper sternum (G1), lower right intercostal space (BE), and lower left intercostal space (G2) to optimize R‐wave detection.

We employed Lorenz plot analysis (Toichi et al. 1997) to calculate autonomic nervous system indices, including the cardiac sympathetic index (CSI) and the cardiac vagal index (CVI). Lorenz plot analysis is particularly useful for analyzing autonomic nervous system activity during craft tasks due to its reduced sensitivity to respiratory components and high overall sensitivity (Gamelin et al. 2006; Penttilä et al. 2001). R–R intervals (RRI) were extracted using Physiozoo (v1.7.1) software, and the presence or absence of artifact or missing data were visually checked during the extraction process. The Lorenz plot is a scatterplot showing the *n*th RRI plotted on the horizontal axis and the *n +* 1‐th RRI plotted on the vertical axis, where *SD1* is the standard deviation in the vertical direction and *SD2* is the standard deviation in the direction parallel to the same line of the distribution. The values of *SD1* and *SD2* were obtained for each participant for the entire duration of the task, the first minute immediately after the task began, and the last minute immediately before the task ended. *L* and *T* are four times the values of *SD1* and *SD2*, respectively, and CSI is obtained as the ratio of *L/T*, while CVI is obtained as the natural logarithm of L*T (Toichi et al. 1997). Change scores for CSI, CVI, and RRI were calculated by subtracting the value from the first minute of the task from the value from the last minute of the task.

### 2.4 Statistical Analysis

Task performance (in seconds), FAA scores, and autonomic data (total CSI, total CVI, total RRI, CSI change, CVI change, and RRI change) were examined for normality by using the Shapiro–Wilk test. Depending on data normality, paired *t*‐tests or Wilcoxon signed‐rank sum tests were used to compare conditions between altruistic and selfish conditions. For all statistical tests, effect sizes were calculated: Cohen's *d* for parametric tests and *r* for non‐parametric tests. The criteria for interpreting effect sizes were as follows: small (*d* = 0.2, *r* = 0.1), medium (*d* = 0.5, *r* = 0.3), and large (*d* = 0.8, *r* = 0.5). This statistical analysis was performed using jamovi (version 2.4.0), and the significance level was set at 5%.

To address the issue of multiple comparisons inherent in our multi‐channel EEG data, we employed distinct correction strategies appropriate for each level of analysis. For sensor‐level analyses involving spectral power across multiple electrodes and frequency bands, we utilized the False Discovery Rate (FDR) correction method within EEGLAB. This approach balances control over Type I errors with statistical power, with the significance level set at a corrected *p* < 0.05. The results were presented by subtracting the average spectrum for each subject and plotting the averaged topography over the frequency range. The frequency bands were set as follows: delta band 1–4 Hz, theta band 4–8 Hz, alpha band 8–13 Hz, beta band 13–30 Hz, and gamma band 30–60 Hz.

For the source‐level CSD and FC analyses, which involve statistical comparisons across thousands of voxels, we transitioned from FDR to a more robust non‐parametric permutation approach within the eLORETA software package. For CSD and FC analyses, we used the eLORETA software package to perform statistical nonparametric mapping (SnPM) (Anderer et al. 1998; Pascual‐Marqui et al. 1999). This method is robust as it does not rely on assumptions of data normality. To address the issue of multiple comparisons across all voxels and frequencies, all statistical tests within eLORETA were corrected using a non‐parametric randomization procedure based on 5000 permutations (Pascual‐Marqui et al. 2002). This comprehensive correction was applied uniformly to all condition comparisons and correlation analyses.

Based on the distribution of corrected *p*‐values derived from this permutation testing, we applied two thresholds for reporting our results. Results with a corrected *p*‐value < 0.05 were considered statistically significant. Additionally, to facilitate future research and hypothesis generation, results with a corrected *p*‐value between 0.05 and 0.10 were reported as “statistical trends” or “exploratory findings.” This two‐tiered approach ensures statistical rigor while maintaining transparency about findings that approached, but did not reach, the conventional significance threshold after stringent correction.

## 3 Results

Three participants were excluded from the final analysis due to deviations from task instructions (*n* = 1) and interruptions caused by thread detachment (*n* = 2), resulting in a final sample of 31 participants (14 males, 17 females; mean age 20.00 ± 1.18 years). We present our findings in three main subsections: (1) behavioral and global EEG measures, (2) CSD and FC, and (3) neural correlations.

### 3.1 Behavioral and Global EEG and Autonomic Measures

#### 3.1.1 Task Performance

We found no significant difference in the time required to complete the task between the altruistic (220.65 ± 55.69 s) and selfish (205.13 ± 56.34 s) conditions (*p* = 0.104) (Table 1).

**TABLE 1:** Comparison of task performance and FAA scores and autonomic activity.

	Altruistic		Selfish		*p*	Effect size
	Mean	SD	Mean	SD		
	Median	IQR	Median	IQR		
Time (s) †	220.65	55.69	205.13	56.34	0.104	0.30
FAA ††	−0.010	0.02	−0.015	0.03	0.031*	0.45
Total CSI ††	2.24	0.88	2.14	1.14	0.232	0.25
Total CVI †	4.28	0.37	4.24	0.37	0.921	0.02
Total RRI (ms) ††	791.53	169.21	797.66	173.77	0.794	0.06
CSI change †	−0.26	0.59	0.11	0.72	0.048*	0.37
CVI change †	−0.04	0.24	0.05	0.17	0.134	0.28
RRI change [ms] †	−12.52	32.06	−10.43	29.80	0.617	0.09

*Note*: †: Mean and standard deviation are presented, *p*‐values are from the paired *t*‐test, and effect sizes are presented as Cohen's *d*. ††: Median and interquartile range are presented, *p*‐values are from the Wilcoxon signed rank test, and effect size is Wilcoxon's *r*. *: *p* < 0.05.

Abbreviations: CSI, cardiac sympathetic index; CVI, cardiac vagal index; FAA, frontal alpha asymmetry; IQR, interquartile range; RRI, R‐R interval; SD, standard deviation.

#### 3.1.2 FAA Score

**FIGURE 2:**
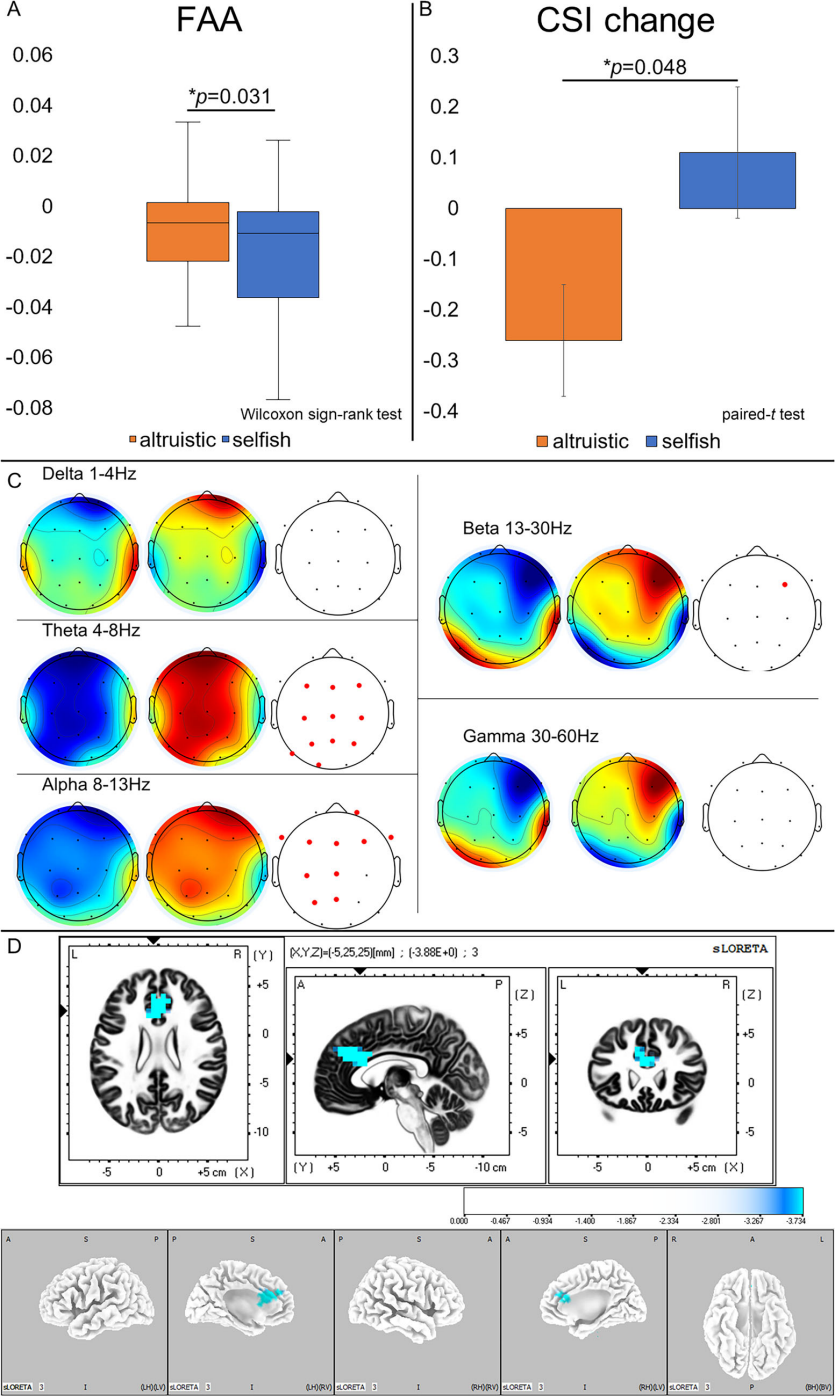
Comparative analysis of neurophysiological measures across conditions. (A) FAA Score Comparison: The box plots show that FAA scores are notably higher in the altruistic condition than in the selfish condition (*p* = 0.031). Whiskers represent minimum and maximum values, and the central line indicates the median. (B) CSI Change Comparison: The bar graph shows a significantly greater negative change in CSI during the altruistic condition than during the selfish condition (*p* = 0.048). Error bars represent the standard error of the mean. (C) Topographic Maps of Cortical Activity Across Frequency Bands: From left to right, the following frequency bands are shown: delta (1–4 Hz), theta (4–8 Hz), alpha (8–13 Hz), beta (13–30 Hz), and gamma (30–60 Hz). For each band, the left map shows activity during the altruistic condition, the center map shows activity during the selfish condition, and the right map shows electrodes with significant differences between the two conditions (*p* < 0.05, red). The results were presented by subtracting the average spectrum for each subject and plotting the averaged topography over the frequency range. The color scale represents spectral power, with red indicating higher power and blue indicating lower power. The rightmost map for each band highlights, in red, electrodes with significant differences between conditions (*p* < 0.05, FDR‐corrected). (D): CSD comparison. The color map displays *t*‐values from the contrast (Altruistic—Selfish), with lighter blue indicating stronger effects. The anterior cingulate cortex exhibited the most pronounced difference between conditions (*p* = 0.073). FAA: frontal alpha asymmetry; CSI: cardiac sympathetic index.

We observed that the altruistic condition (−0.010 [−0.022 to 0.001]) yielded significantly higher FAA scores compared to the selfish condition (−0.015 [−0.037 to 0.003]) (*W* = 358.50, *p* = 0.031, *r* = 0.45) (Figure 2A).

#### 3.1.3 Cortical Activity across Frequency Bands

In the theta band, we observed significantly lower activity in the altruistic condition for Fz, F3, F4, Cz, C3, C4, Pz, P3, P4, O1, and T5 electrodes (*p* < 0.05). In the alpha band, we found significantly reduced activity in the altruistic condition for Fp2, Fz, F3, F4, F7, F8, Cz, C3, Pz, and P3 electrodes (*p* < 0.05). For the beta band, we identified significantly lower in the F4 region during the altruistic condition (*p* < 0.05). We detected no significant differences in the delta and gamma bands (*p* > 0.05) (Figure 2C).

#### 3.1.4 Autonomic Activity

Our analysis showed that the CSI change was significantly more negative in the altruistic condition (−0.26 ± 0.59) compared to the selfish condition (0.11 ± 0.72) (*t*(30) = 2.06, *p* = 0.048, Cohen's *d* = 0.37) (Figure 2B). We observed no other significant differences or trends (*p* > 0.10).

### 3.2 CSD and FC

Using eLORETA, we identified a trend toward lower alpha activation in the ACC in the altruistic condition compared to the selfish condition (*p* = 0.073) (Figure 2D). We observed no significant differences in FC between the conditions.

### 3.3 Neural Correlations

#### 3.3.1 CSD and Neurophysiological Indices

**FIGURE 3:**
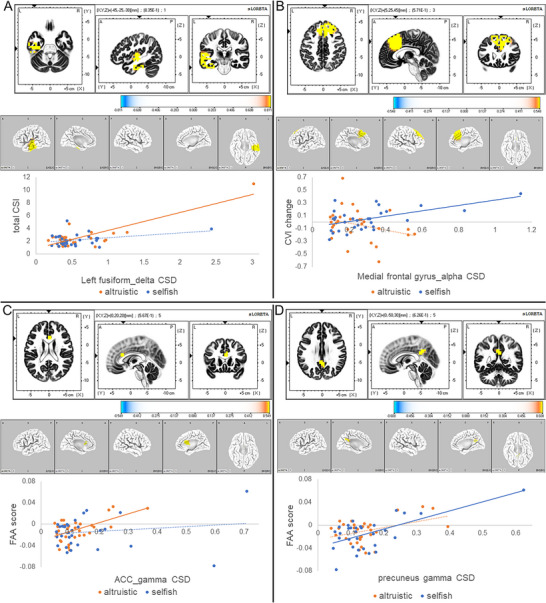
Correlation Between CSD and Neurophysiological Indices. (A) Correlation Between Total CSI and Left Fusiform Delta Activity. The statistical map shows the *t*‐values for the positive correlation. Yellow highlights the region with the strongest trend. The minimum *p*‐value was 0.070 for the altruistic condition and greater than 0.10 for the selfish condition. The scatterplot illustrates the correlation between left fusiform delta CSD and total CSI for the altruistic (orange) and selfish (blue) conditions. (B) Correlation Between Change in CVI and Alpha Activity in the Medial Frontal Gyrus. The statistical map shows the *t*‐values for the positive correlation. Yellow highlights the region with the strongest trend. The minimum *p*‐value was 0.10 for the altruistic condition and 0.066 for the selfish condition. The scatterplot illustrates the correlation between alpha CSD in the medial frontal gyrus and change in CVI for the altruistic (orange) and selfish (blue) conditions. (C) Correlation between FAA score and ACC gamma activity. The statistical map shows the *t*‐values for the positive correlation, with yellow highlighting the region with the strongest trend. The minimum *p*‐value was 0.071 for the altruistic condition and > 0.10 for the selfish condition. The scatterplot shows the correlation between ACC gamma CSD and FAA score for the altruistic and selfish conditions. (D) Correlation Between FAA Score and Precuneus Gamma Activity. The statistical map shows *t*‐values for positive correlations, with yellow highlighting regions with the strongest trends. The minimum *p*‐value was greater than 0.10 for the altruistic condition and 0.029 for the selfish condition. The scatterplot illustrates the correlation between precuneus gamma CSD and FAA scores in the altruistic (orange) and selfish (blue) conditions. ACC: anterior cingulate cortex; CSD, current source density; CSI, cardiac sympathetic index; CVI, cardiac vagal index; FAA, frontal alpha asymmetry.

1. In the altruistic condition, we observed a positive correlation trend (*p* = 0.070) between total CSI and delta activity in the left fusiform gyrus. We did not observe this correlation in the selfish condition (Figure 3A).
2. In the selfish condition, we identified a positive correlation trend (*p* = 0.066) between CVI change and alpha activity in the medial frontal gyrus. This trend was absent in the altruistic condition (Figure 3B).
3. Our exploratory analysis revealed a marginal association between FAA scores and gamma activity in the ACC (*p* = 0.071; Figure 3C) during the altruistic condition. Conversely, in the selfish condition, we found a significant positive correlation (*p* = 0.029) between FAA scores and gamma activity in the precuneus (Figure 3D).

#### 3.3.2 FC and Neurophysiological Indices

**FIGURE 4:**
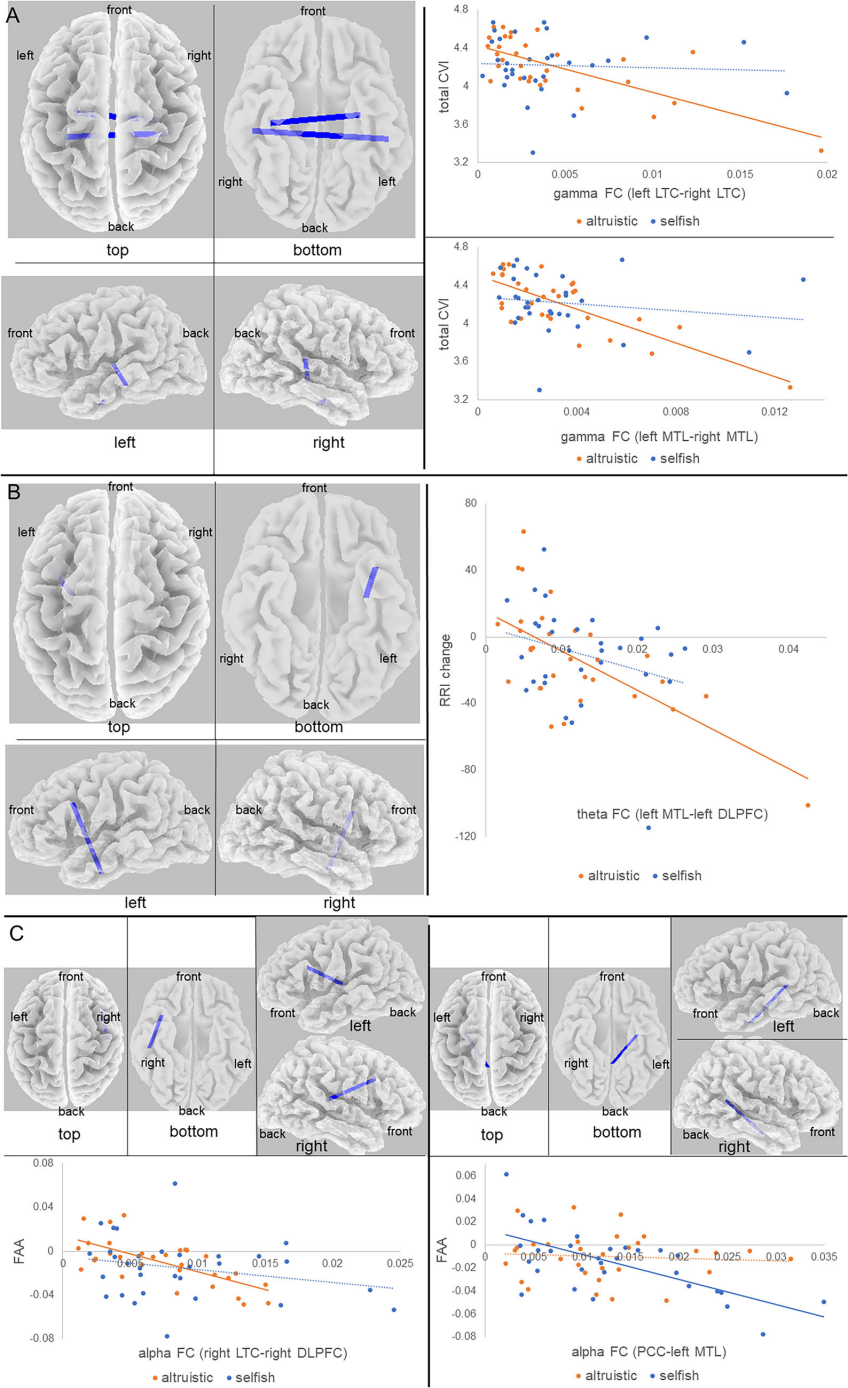
Correlation Between FC and Neurophysiological Indices. (A) Correlations Between Total CVI and Gamma‐Band FC. The blue lines in the brain model depict significant negative correlations in the altruistic condition (*p* = 0.002). The scatterplots show the relationship between total CVI and gamma‐band connectivity in the left and right LTC and MTL networks in the altruistic (orange) and selfish (blue) conditions. (B) Correlations Between RRI Change and Theta‐Band FC. The brain model shows a significant negative correlation between the left MTL and left DLPFC in the altruistic condition (*p* = 0.048). The scatterplot illustrates the relationship between theta connectivity between the left MTL and left DLPFC and RRI change in both conditions. (C) Correlations Between FAA Scores and Alpha‐Band FC. The left panels show a significant negative correlation between the right LTC and the right DLPFC in the altruistic condition (*p* = 0.007). The right panels show a significant negative correlation between the PCC and the left MTL in the selfish condition (*p* = 0.035). The scatterplots display the correlation between FAA scores and alpha connectivity for both conditions. The scatterplot on the left corresponds to the right LTC‐DLPFC network, and the scatterplot on the right corresponds to the PCC‐MTL network. CVI, cardiac vagal index; DLPFC, dorsolateral prefrontal cortex; FAA, frontal alpha asymmetry; FC, functional connectivity; LTC, lateral temporal cortex; MTL, medial temporal lobe; PCC, posterior cingulate cortex; RRI, R‐R interval.

1. In the altruistic condition, we observed significant negative correlations (*p* = 0.002) between total CVI and gamma‐band FC in both the left‐right lateral temporal cortex (LTC) and the left‐right medial temporal lobe (MTL). These correlations were not present in the selfish condition (Figure 4A). This suggests that higher parasympathetic activity is associated with reduced interhemispheric temporal lobe connectivity during altruistic behavior.
2. We also found a significantly negative correlation (*p* = 0.048) between RRI change and theta‐band FC between the left MTL and left DLPFC in the altruistic condition. This correlation was not significant in the selfish condition (Figure 4B). This indicates that higher heart rate is associated with reduced connectivity between memory and executive function areas during altruistic tasks.
3. In the altruistic condition, we identified a significant negative correlation (*p* = 0.007) between FAA scores and alpha‐band FC between the right LTC and right DLPFC. In the selfish condition, we found a significant negative correlation (*p* = 0.035) between FAA scores and alpha‐band FC between the posterior cingulate cortex (PCC) and left MTL (Figure 4C). These findings suggest that frontal asymmetry is associated with distinct patterns of cortical connectivity in altruistic versus selfish contexts.

## 4 Discussion

This study investigated the neurophysiological underpinnings of altruistic and selfish motivations using simultaneous EEG and ECG. We found that the altruistic condition showed significantly higher FAA scores, suggesting greater approach motivation, consistent with altruistic motivation activating social reward systems and fostering positive affect (Allen et al. 2018; Coan and Allen 2003, 2004; Izuma et al. 2010; Shangguan et al. 2023). Additionally, the altruistic condition exhibited significantly lower CSI, indicating a stress‐reducing effect associated with performing a task for someone else. This reduced sympathetic activity aligns with previous research on altruism promoting psychological well‐being (Cornelissen and Fagard 2005; Elsherbiny 2022; Lambert et al. 2015; L. Li et al. 2023). The lack of significant differences in parasympathetic activity (CVI) suggests that altruistically motivated tasks may primarily inhibit sympathetic activity, potentially due to the higher responsiveness of the sympathetic system (Moini et al. 2024). This profile of increased left frontal activity and reduced sympathetic arousal associated with positive affective states supports the potential use of altruistically motivated tasks in therapeutic and rehabilitation settings.

eLORETA analysis revealed distinct cortical activity patterns distinguishing the two motivational states. Most notably, we observed a trend toward lower alpha activity in the ACC during the altruistic condition, suggesting increased activation in this key hub for emotion processing and incorporating others' perspectives (Fellows and Farah 2005; Lavin et al. 2013; Mohanty et al. 2007; Apps et al. 2012; Lockwood et al. 2018). This source‐level finding was complemented by sensor‐level results showing widespread alpha and theta reduction. The central and parietal regions were associated with spatial processing and body image (Galati et al. 2001; Moayedi et al. 2021), and the occipital region with visual processing areas (Braddick 2015). The present findings suggest increased attention to spatial relationships between self and others, as well as to bodily states and visual attention, which is consistent with enhanced empathic processes underlying the altruistic mindset. This may also involve mental imagery of the recipients, even if not physically present during the task. The observed decrease in theta activity during the altruistic condition is particularly intriguing. Theta activity in the medial frontal cortex is associated with attentional control and executive function (Wen et al. 2023; Brandmeyer and Delorme 2020; Ishii et al. 1999). Our results suggest a shift from egocentric to other‐oriented processing (Jiang et al. 2021; Laquitaine et al. 2024) during the altruistically motivated task. Furthermore, in the beta band, significantly lower activity was observed in the frontal right area (F4) during the altruistic condition. The right frontal area is associated with emotional and inhibitory control (Gavazzi et al. 2023; Wyczesany et al. 2024), and this finding may reflect changes in neural mechanisms to inhibit selfish responses and promote more other‐oriented processing when acting altruistically. The F4 electrode location can also be interpreted as capturing activity in the right DLPFC (Im et al. 2019). Given the repetitive transcranial magnetic stimulation (rTMS) suppressing right DLPFC activity has been reported to have antidepressant effects (Lefaucheur et al. 2020), it is possible that positive mental states were induced in the altruistic condition.

To advance our understanding of the cortical mechanisms underlying FAA, we performed an exploratory analysis examining its correlation with CSD, particularly in the context of altruistically motivated behavior. Uncovering these neural correlates is crucial not only for elucidating the neurophysiological basis of positive affect but also for informing future therapeutic strategies like neurofeedback. In this analysis, we found a distinct pattern of correlations. In the altruistic condition, we observed a positive correlation trend between FAA scores and gamma activity in the ACC. Given that the ACC is a key hub for cognitive control and emotion processing (Herrmann et al. 2004; Jensen et al. 2007), this exploratory finding suggests that the positive affect associated with altruistic motivation may be linked to the cognitive control required to integrate another's perspective. Conversely, in the selfish condition, FAA scores significantly correlated with gamma activity in the precuneus, a region central to self‐referential thought. This sharp contrast in neural correlates—ACC for altruism versus precuneus for selfishness—provides a compelling, albeit preliminary, neural signature differentiating these two motivational states. While these findings require replication, they offer valuable hypotheses for future research and potential targets for interventions aimed at enhancing prosocial behavior. FAA scores in the altruistic condition also showed a significant negative correlation with alpha coupling in the right LTC‐right DLPFC. This suggests that lower FAA (less positive affect) is linked to stronger alpha coupling (cortical inhibition) in regions involved in nonverbal processing and emotion regulation (Davidson 1988; Peterson and Voytek 2017; Chen et al. 2019; Golby et al. 2001; Rice et al. 2015; Keuper et al. 2018). For the selfish condition, FAA negatively correlated with alpha coupling in the PCC‐left MTL network, implicated in autobiographical memory (Roehri et al. 2022; Kaboodvand et al. 2018). These findings suggest that social cognition, including nonverbal processing of information about others, is important for eliciting positive emotions in altruistic motivation, whereas memories about the self are important for selfish motivation.

Our results also illuminated intricate relationships between autonomic activity and cortical CSD/FC. In the altruistic condition, a positive correlation trend between total CSI and left fusiform gyrus delta activity suggests that altruistic motivation toward familiar others may contribute to reduced sympathetic activity, given the fusiform's role in face recognition and social cognition (Kim et al. 2019; Ngo and Born 2019; George et al. 1999; W. Li et al. 2021). Conversely, in the selfish condition, a trend for positive correlation between CVI change and medial frontal gyrus alpha activity suggests that stronger self‐focused thoughts may be linked to reduced parasympathetic activity (Davidson 1988; Peterson and Voytek 2017; Burden et al. 2021; Levorsen et al. 2024). Furthermore, in the altruistic condition, total CVI negatively correlated with gamma coupling between bilateral LTC and MTL, indicating that increased memory processing in these regions, perhaps related to familiar others, is associated with reduced parasympathetic activity (Dalton et al. 2016; Gotts et al. 2013; Griffiths et al. 2023). Lastly, RRI change in the altruistic condition negatively correlated with theta coupling between the left MTL and left DLPFC. This suggests that less demanding recognition processes for familiar others may be associated with increased parasympathetic activity, as this coupling is critical for recognition memory (Welke et al. 2023).

The integration of EEG and ECG data in this study provides a unique opportunity to explore the complex interplay between cortical activity and autonomic regulation during tasks driven by altruistic versus selfish motivations. Our findings can be interpreted within the framework of the neurovisceral integration model proposed by Thayer and Lane (2000, 2009). This model posits that the prefrontal cortex, particularly the mPFC and ACC, exerts top‐down control over subcortical structures, including those regulating autonomic function. In line with this model, our observed correlations between FAA scores (reflecting prefrontal activity) and autonomic indices (CSI and CVI) suggest a direct link between cortical emotional processing and physiological arousal states. Furthermore, the observed correlations between the activity of specific areas and autonomic measures align with the concept of “central autonomic networks” (Beissner et al. 2013). These networks involve cortical, limbic, and brainstem structures that coordinate autonomic, endocrine, and behavioral responses. The differential patterns of correlation observed in altruistic versus selfish conditions may reflect distinct modes of neurovisceral integration, potentially related to the social‐cognitive demands of each condition. However, it is important to note that these interpretations are speculative and require further validation. Future studies should employ more sophisticated analytical techniques, such as dynamic causal modeling or Granger causality analysis, to elucidate the directional relationships between cortical activity and autonomic regulation during socially motivated actions.

The findings of this study have several potential clinical applications, although it is crucial to emphasize that these are speculative and require further validation in clinical settings. First, neurofeedback training promoting an altruistic mindset could be incorporated into therapeutic practice using FAA and specific EEG patterns as indicators. Second, the neurophysiological changes revealed in this study could serve as efficacy measures for various treatments, objectively evaluating improvements in social behavior and stress response. Third, the association between altruistic motivation and FC between specific brain regions may allow for more effective design of group therapy dynamics and development of methods to optimize interactions among participants. However, caution should be exercised when translating these results directly into clinical applications, and further validation is needed to generalize the results from healthy subjects to clinical populations. It will be important to investigate the clinical significance and plasticity of these neurophysiological changes through studies in clinical groups and intervention studies.

Several limitations should be considered when interpreting our findings. First and foremost, it is crucial to acknowledge that our paradigm measured the neurophysiological correlates of a motor task performed with altruistic intent, not a direct social interaction. Therefore, our findings pertain to the internal motivational and affective states associated with altruism, and caution should be exercised when generalizing these results to complex, real‐world social behaviors. The correlational nature of our data and the relatively small sample size (*n* = 31) prevent definitive causal conclusions and limit generalizability, despite our a priori power analysis. Future research should employ larger, more diverse samples and consider higher density EEG systems for improved spatial resolution. It is important to note that some results were statistical trends (0.05 < *p* < 0.10) even after multiple comparison correction, requiring cautious interpretation and validation in future studies. Our study design does not allow conclusions on causality; therefore, interventional approaches or longitudinal designs are necessary to elucidate direct causal relationships. As a short‐term laboratory observation, our study may not fully capture long‐term dynamics of altruistic behavior. Future research should investigate long‐term effects, habituation, and behavioral measures in actual social interactions across diverse cultural and age groups.

Despite these limitations, our findings contribute to understanding prosocial motivation and offer perspectives for clinical practice. Potential applications include neurofeedback for fostering altruistic mindsets, utilizing neurophysiological changes as treatment efficacy measures, and informing group therapy design. Further validation in clinical populations is crucial to translate these speculative applications.

## 5 Conclusion

This study offers preliminary insights into the neurophysiological basis of tasks performed for altruistic or selfish motivations. Tasks performed with altruistic intent are characterized by increased frontal lobe asymmetry, suggesting increased positive emotion and decreased sympathetic activity. These tasks are also associated with specific patterns of cortical and network activity related to other‐oriented processing, social cognition, and bottom‐up attentional mechanisms. Although the preliminary neural associations require replication, the core neurophysiological and autonomic findings reveal significant physiological differences between altruistic and selfish motivated conditions.

In conclusion, this study contributes to our understanding of the neural architecture supporting altruism and opens new avenues for future research. The implications of these findings extend beyond neuroscience and could inform practices in psychology, psychiatry, and rehabilitation sciences. However, it is crucial to approach these potential applications with caution. Future research should elucidate the causal mechanisms underlying the correlations observed in this study and investigate long‐term effects to archive a deeper understanding of the neural basis of altruistic behavior.

## Author Contributions

**Junya Orui**: Conceptualization, Methodology, Investigation, Formal Analysis, Supervision, Project Administration, Writing – Original Draft, Writing – Review & Editing. **Keigo Shiraiwa**: Conceptualization, Methodology, Investigation, Formal Analysis, Writing – Review & Editing. **Takao Inoue**: Conceptualization, Methodology, Writing – Review & Editing. **Masaya Ueda**: Investigation, Formal Analysis, Writing – Review & Editing. **Keita Ueno**: Investigation, Formal Analysis, Writing – Review & Editing. **Yasuo Naito**: Formal Analysis, Writing – Review & Editing. **Ryouhei Ishii**: Conceptualization, Methodology, Formal Analysis, Writing – Original Draft, Writing – Review & Editing.

## Peer Review

The peer review history for this article is available at https://publons.com/publon/10.1002/brb3.70747.

## Data Availability

The data that support the findings of this study are available from the corresponding author upon reasonable request.
